# Construction of genetically encoded biosensors for monitoring cytosolic and mitochondrial H_2_O_2_ in response to nanozymes in THP-1 cells

**DOI:** 10.52601/bpr.2025.250008

**Published:** 2025-10-31

**Authors:** Tao Wang, Mengfan Yu, Chenshuo Ren, Fan Yang, Tao Wen, Xian-En Zhang, Haoan Wu, Yu Zhang, Dianbing Wang, Haiyan Xu

**Affiliations:** 1 Institute of Basic Medical Sciences, Chinese Academy of Medical Sciences & Peking Union Medical College, Beijing 100005, China; 2 Key Laboratory of Biomacromolecules (CAS), National Laboratory of Biomacromolecules, CAS Center for Excellence in Biomacromolecules, Institute of Biophysics, Chinese Academy of Sciences, Beijing 100101, China; 3 University of Chinese Academy of Science, Beijing 100049, China; 4 Faculty of Synthetic Biology, Shenzhen University of Advanced Technology, Shenzhen 518055, Guangdong, China; 5 State Key Laboratory of Bioelectronics, Jiangsu Key Laboratory for Biomaterials and Devices, School of Biological Science and Medical Engineering & Collaborative Innovation Center of Suzhou Nano Science and Technology, Southeast University, Nanjing 211189, China

**Keywords:** H_2_O_2_, Nanozymes, Oxidoreductase, Biosensors

## Abstract

Intracellular H_2_O_2_ levels are tightly regulated and can be modulated by various stimuli. A variety of nanozymes have been revealed with the ability to catalyze substrates of oxidoreductases, mostly including peroxidase (POD), superoxide dismutase (SOD) and catalase (CAT), and some of them display multienzyme-like properties, which make them highly attractive for biomedical applications. However, the specific manifestations of nanozyemes within cells remain challenging to predict and detect. In this study, we developed a real-time, dynamic, and highly sensitive live-cell biosensor by expressing HyPer7 probe in the cytosol and mitochondria to monitor the cytosolic and mitochondrial H_2_O_2_ dynamics in a leukemia cell line THP-1. The successful expression of the probes in the cytosol and mitochondria was confirmed using confocal fluorescence microscopy. When the THP-1 cells were exposed to exogenous H_2_O_2_, the fluorescence intensity at 525 nm upon excitation with 405 nm lasers (referred to as F405) decreased, while that upon excitation with 488 nm lasers (referred to as F488) increased. Using this biosensor, we examined the dynamics of cytosolic and mitochondrial H_2_O_2_ in response to Daunorubicin, Fe_3_O_4_ nanozyme with Polyetherimide (PEI)- or Dextran (Dex)-modification, and Prussian blue nanozyme with different diameters. Results indicated that the particle size of PBNPs and surface modification of Fe_3_O_4_ play critical roles in their intracellular effects on the aspect of H_2_O_2_ modulation. The live-cell biosensors thus provide a powerful tool for detecting the variations of cytosolic and mitochondrial H_2_O_2_ in response to nanozymes, thereby facilitating a better understanding of the biological effects of nanozymes and their potential biomedical applications.

## INTRODUCTION

Reactive oxygen species (ROS), which originate from molecular oxygen and are produced through redox reactions or electronic excitation, have attracted widespread research attention due to their contentious effects (Sies *et al.* 2022). ROS are believed to play a role in oxygen toxicity owing to their heightened chemical reactivity. Moreover, they act as intracellular signaling molecules, taking part in various physiological and pathological processes (D'Autréaux and Toledano 2007; Sies *et al.* 2024). In recent years, it has become evident that using ROS as a blanket term is somewhat imprecise, given that each type of reactive oxygen species possesses distinct properties and functions (Murphy *et al.* 2022; Sies and Jones 2020).

Among the diverse ROS molecules, hydrogen peroxide (H_2_O_2_) stands out as the primary ROS involved in the redox regulation of biological activities. H_2_O_2_ serves as a versatile and pleiotropic physiological signaling agent, functioning as a second messenger in biological processes by reversibly oxidizing specific protein thiolates (Sies and Jones 2020). The intracellular concentration of H_2_O_2_ is maintained in the low nanomolar range (approximately 1–100 nmol/L) and is under tight control (Parvez *et al.* 2018). H_2_O_2_ is produced from various sources within cells, including specific enzymatic sources such as NADPH oxidases (NOXs) (Bedard and Krause 2007), as well as the mitochondrial electrons transport chains (Murphy 2009), and removed via several intrinsic anti-oxidant small molecules and enzymes, including the thioredoxin system and the glutathione system. Other oxidoreductase can also modulate the intracellular H_2_O_2_ concentration, including peroxidases (POD), superoxide dismutase (SOD), and catalase (CAT). These properties modulated ROS towards different directions, for example, POD catalyzes the oxidation of substrates in the presence of peroxides (mostly H_2_O_2_ with a few as organic hydroperoxides) (Jiang *et al.* 2019), therefore consuming hydrogen peroxide to generate other oxides, while SODs disproportionates superoxide radicals into oxygen and H_2_O_2_ (Jiang *et al.* 2019) to increase the concentration of hydrogen peroxide, and catalase accelerates the dismutation of H_2_O_2_ into water and oxygen (Jiang *et al.* 2019), therefore scavenging local H_2_O_2_.

It has been well documented that nanozymes hold the ability to catalyze specific biochemical reactions, showing effects similar to natural enzymes (Ren *et al.* 2022). The most commonly exhibited properties of nanozymes were oxidoreductase-like activity (Jiang *et al.* 2019), including peroxidases (POD), superoxide dismutase (SOD), and catalase (CAT). Many nanozymes, such as metal (Guan *et al.* 2024), metal oxides (Gao *et al.* 2007) and Prussian Blue nanoparticles (Zhang *et al.* 2016), even exhibit multienzyme-like properties in modulating ROS. However, the specific manifestations of nanozymes within cells remain difficult to predict and detect, because different physicochemical properties lead to differences in intracellular distributions and predominant activities. Therefore, it would be beneficial to monitor the dynamics of H_2_O_2_ of certain organelles for understanding the intracellular activity of nanozymes, especially those that exhibit oxidoreductase-like activities.

Monocytes as part of the innate immune system are one of the first immune cells that are at the sites of infections contributing to pathogen defense with phagocytosis, cytokine and reactive oxygen species (ROS) production. THP-1 monocytes, isolated from the peripheral blood of a boy with acute myeloid leukemia (Tsuchiya *et al.* 1980), are widely used as model systems for immunomodulation studies including drug and natural product testing (Schultze *et al.* 2017). At the same time, THP-1 is a cell line widely used in the investigations of acute myeloid leukemia (Lübbert *et al.* 1992).

Genetically encoded fluorescent protein sensors have provided major advances in cellular H_2_O_2_ detection (Bilan and Belousov 2018; Morgan *et al.* 2016). These probes contain a dithiol switch that changes the overall fluorescence of the probe depending on its oxidation status. High sensitivity and specificity for H_2_O_2_ have been achieved by coupling a redox-sensitive green fluorescent protein (GFP) mutant to a H_2_O_2_-sensitive thiol protein, such as oxyR (HyPer series) (Bilan and Belousov 2018), or to a peroxidase such as Orp1 or TSA2 (roGFP2-based probes) (Morgan *et al.* 2016). Among the several biosensors, HyPer7 is a pH-insensitive, genetically encoded H_2_O_2_ reporter, which consists of a cyclically permutated GFP with N- and C-terminal OxyR-RD domain derived from *Neisseria meningitidis* (Pak *et al.* 2020). Following oxidation by H_2_O_2_, HyPer7 forms an intramolecular disulfide bridge that alters the excitation spectra, and the maximum excitation of the HyPer7 chromophore shifts from 405 nm in the reduced state to 488 nm in the H_2_O_2_-oxidized state (Pak *et al.* 2020; Yang *et al.* 2023). Here, we constructed real-time, dynamic, and highly sensitive live-cell biosensors to monitor the cytosolic and mitochondrial H_2_O_2_ dynamics in a leukemia cell line THP-1, utilizing Hyper7 fused with subcellular localization guide peptides mitochondria localization sequence (MLS) and nuclear exclusion sequence (NES) to monitor cytosolic and mitochondrial H_2_O_2_ dynamics respectively in THP-1 cells, aiming to provide a powerful tool for detecting cytosolic and mitochondrial H_2_O_2_ in response to nanozymes.

## MATERIALS AND METHODS

### Cell culture

THP-1 cells were purchased from the Cell Resource Center of the Chinese Academy of Medical Sciences (Beijing, China) and cultured in modified RPMI medium (HyClone, Cytiva, Logan, Utah, USA) supplemented with 10% fetal bovine serum (FBS, Gibco, Thermo Fisher Scientific, Carlsbad, CA, USA), and 100 μg/mL penicillin-streptomycin (HyClone) in a humidified atmosphere of 5% CO_2_ at 37°C. HEK 293T cells were cultured in DMEM (Gibco) supplemented with 10% FBS.

### Construction of cell lines

HyPer7 was fused at the N–terminal of the protein with nuclear export sequence (NES, NSNELALKLAGLDINK) and mitochondrial localization sequence (MLS, MSVLTPLLLRGLTGSARRLPVPRAKIHSL) to express the sensor in the cytosol (CytoHyPer7), mitochondria (MitoHyPer7) of the cell, respectively. In brief, The THP-1 cells constructively expressed CytoHyPer7 and MitoHyPer7 were established by infecting with lentiviral carry the sequences. To obtain lentiviral, the sensor vectors (pLVX-NES-HyPer7, pLVX-MLS-HyPer7) together with three lentiviral packaging vectors (pLPI, pLPII, and pLPVSVG) were used to transfect HEK 293T cells at 50%–60% confluency by Lipofectamine 3000 (Invitrogen, USA) according to the manufacturer’s instructions. The culture supernatant containing recombinant lentivirus was harvested after 72 h. Then THP-1 cells were seeded in six-well culture plates for lentiviral infection in the presence of 4 μg/mL of polybrene (Macgene, Beijing, China) followed by centrifugation at 1000*g* for 1 h at 37°C. Following the lentivirus infection, cells were cultured for 1 week in media containing 3 μg/mL puromycin. Afterward, the fluorescent cells were sorted by FACS Aria IIIu (BD Biosciences, Franklin Lakes, NJ, USA).

### Nanozymes

Nanozymes used in this study included Prussian Blue Nanoparticles (PBNPs) and Fe_3_O_4_ nanoparticles, PtNPs, Au@Pt MnO_2_, and MnBTC. PBNPs of 3.4 nm, referred to as ultrasmall Prussian Blue Nanoparticles (USPBNPs) were synthesized according to the procedure described previously (Qin *et al.* 2020). Briefly, to prepare PBNPs, 0.75 g of PVP and 0.0275 g of K_3_[Fe(CN)_6_] were dissolved in 10 mL of ethanol solution. After stirring at room temperature for half an hour, the mixture was heated at 80°C for 20 h. The blue product was collected by centrifugation and washed several times with double-distilled water (ddH_2_O). PBNPs, PEI- and Dex-modified Fe_3_O_4_ nanoparticles were purchased from Nanjing NanoEast Biotech Co. LTD. Transmission electron microscopy (TEM) and Dynamic Light Scatter (DLS) were utilized to characterize the shape, size, and Zeta potential of the nanoparticles, TEM images of Fe_3_O_4_ were provided by the supplier. PtNPs and Au@Pt were synthesized by the methods described previously (Wen *et al.* 2020). MnO_2_ and MnBTC were kindly gifted by Prof. Lianbing Zhang (Chen *et al.* 2024).

### Fluorescence microscope

Fluorescence microscopes were utilized to observe the intracellular location of HyPer7. For THP-1-Mito-HyPer-7, the cells were stained with 200 nmol/L MitoTracker® Red CMXRos (#M7512, Invivogen) for 20 min according to the instructions before fixing. Both cells were then fixed with 1 mL of 100% pre-cooled methanol for 10 min and washed with PBS. The cells were then resuspended with 1 mL of PBS, and 200 μL of the cell suspension was subjected to cytospin. The cells were mounted with a mounting medium containing DAPI (#ZLI-9556, Zhongshan Golden Bridge) and covered with a coverslip for observation. The slides were observed and photographed using a confocal microscope (Leica TCS SP8 STED, Leica) under the conditions of *Ex*/*Em* = 350/450 nm (For DAPI), 488/525 nm (For HyPer7), and 577/602 nm (For MitoTracker® Red).

### Flow cytometry

To detect the responsiveness of the biosensors to exogenous H_2_O_2_, THP-1-CytoHyPer7 cells and THP-1-MitoHyPer7 cells with a density of 4 × 10^5^ cell/mL were treated with H_2_O_2_ (10011218, Sinopharm Chemical Reagent Co., Ltd.) at concentrations ranging from 1 μmol/L to 400 μmol/L for 2 min. The fluorescence in the cells was detected by flow cytometry (CytoFLEX, Beckman Coulter) using 405 and 488 nm as excitation lights and collecting the emission light through 525/50 nm and 530/30 nm bandpass filters, respectively. Imaging flow cytometry (ImageStream^X^ MarkⅡ, Merk) was also used to photograph the emission fluorescence at 525 nm upon 488 nm excitation after being incubated with 100 μmol/L H_2_O_2_.

To monitor the dynamics of cytosolic and mitochondrial H_2_O_2_ induced by chemotherapeutics reagents and nanozymes, THP-1-CytoHyPer7 cells and THP-1-MitoHyPer7 cells with a density of 4 × 10^5^ cell/mL were incubated with different concentrations of chemotherapeutics reagents and nanozymes. After co-incubation for 6, 24, and 48 h, the cells were collected by centrifugation, washed once with PBS, and detected by flow cytometry. The experimental data were analyzed using FlowJo (V10).

### Statistics

All data were expressed as the mean ± standard deviation (SD) for at least triplicate experiments. Statistical analysis was performed in Graphpad Prism 8.3.0. To compare the means of three or more groups defined by one factor, One-way ANOVA was employed and followed by Dunnett post-hoc test to compare the means of a prespecified pair of columns. *P* < 0.05 is considered statistically significant.

## RESULTS AND DISCUSSION

### Cytosolic and mitochondrial localization of Hyper7 probe in THP-1 cells

**Figure 1:**
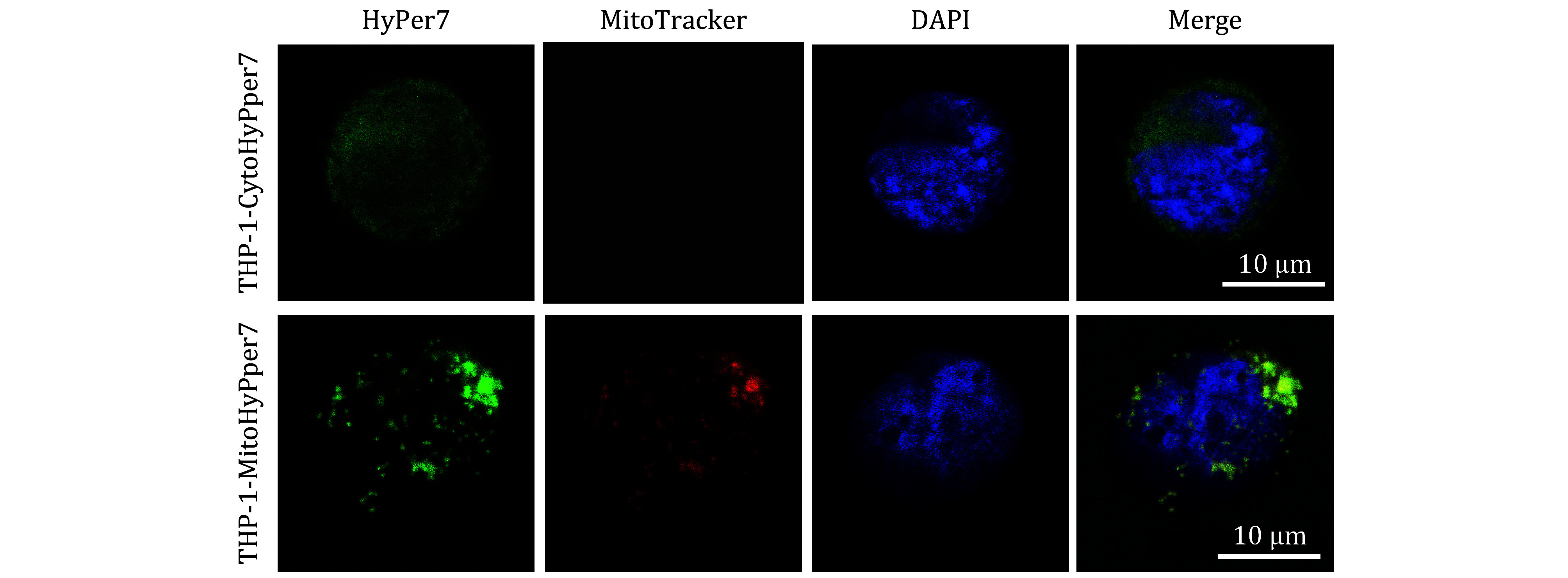
Targeting expression of HyPer7 probe in cytosol and mitochondria. Scale bar = 10 μm

Hyper7 has been widely employed as H_2_O_2_ biosensors in model organisms including yeasts (de Cubas *et al.* 2021; Kritsiligkou *et al.* 2021, 2023), *Arabidopsis thaliana* (Dopp *et al.* 2023), *Mus musculus* (Kano *et al.* 2024; Li *et al.* 2022), Zebrafish *Danio rerio* (Sergeeva *et al.* 2025), as well as cell lines in culture including human umbilical vein endothelial cells (HUVEC) (Jacobs *et al.* 2022; Waldeck-Weiermair *et al.* 2022), mouse hepatocytes (AML12 cells) (Shashkovskaya *et al.* 2023), hippocampal neurons (Kotova *et al.* 2023), human iPCs derived spheroids (Usatova *et al.* 2024). In this study, we applied Hyper7 to monitor the dynamics of cytosolic and mitochondrial H_2_O_2_ in THP-1 cells. The cells expressing Cyto-Hyper7 (upper column in Fig. 1) exclusively exhibited a uniform cytosolic distribution of fluorescent protein sensor signals (green in Fig. 1) compared to the DAPI-stained nucleus (blue in Fig. 1). Mito-Hyper7 (down column in Fig. 1) was intended to target the mitochondria and colocalize with Mito-Tracker Red (red in Fig. 1), and the PCC between the Mito-HyPer7 probe and MitoTracker signal was recorded as 0.90, indicating a collocation. These images clearly confirmed the correct distribution of Cyto-HyPer7 and Mito-HyPer7 in the cytosol and mitochondria, and the successful construction of the biosensors.

### Responsiveness of the biosensors to exogenously added hydrogen peroxide

**Figure 2:**
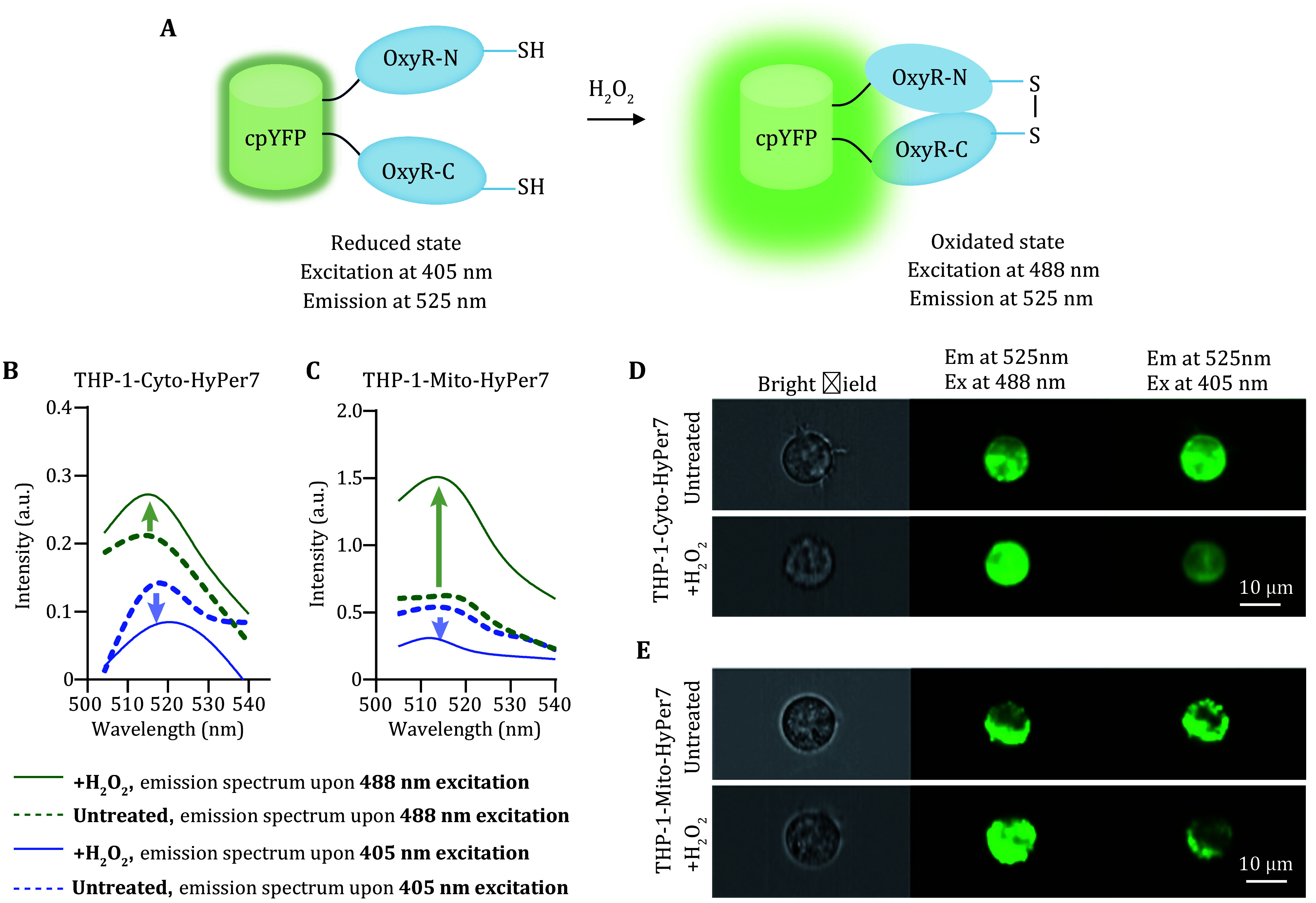
Fluorescence shift of the biosensors in response to exogenously adding H_2_O_2_. **A** Illustration of the mechanism of Hyper7 probe in response to H_2_O_2_. **B**,**C** Emission spectrum of THP-1-CytoHyPper7 cells (**B**) and THP-1-MitoHyPper7 cells (**C**) upon 405 nm (purple lines) and 488 nm (green lines) excitation, the dotted line indicated untreated group, while the solid line indicated cells co-incubation with H_2_O_2_. **D**,**E** Fluorescence at 525 nm upon 488 nm excitation and 405 nm excitation in THP-1-CytoHyper7 cells (**D**) and THP-1-MitoHyper7 cells (**E**), captured by imaging flow cytometry, scale bar =10 μm

H_2_O_2_ can diffuse from extracellular space into cytosol and further into mitochondria (Pak *et al.* 2020). To verify whether the biosensors can respond to the perturbation of H_2_O_2_, we added exogenously H_2_O_2_ to the culture medium, and results showed that externally added H_2_O_2_ caused a detectable oxidation of both probes. Upon oxidation, the excitation spectra of HyPer7 changed with a decrease at 405 nm and an increase of the 488 nm peak, while the emission spectra are similar in both states, peaking at 525 nm (Fig. 2A). Therefore, we detected fluorescence upon the excitation with lasers of 405 nm (referred to as F405) and 488nm (referred to as F488). It was shown that the fluorescence upon 405 nm excitation was reduced while that upon 488 nm was increased when the cells were incubated with exogenous H_2_O_2_ (Figs. 2B and 2C). Images acquired from the imaging flow cytometry (Figs. 2D and 2E) supported the results of flow cytometry, showing that the fluorescence of the cells exposed to 100 μmol/L H_2_O_2_ was brighter than that of the control cells.

**Figure 3:**
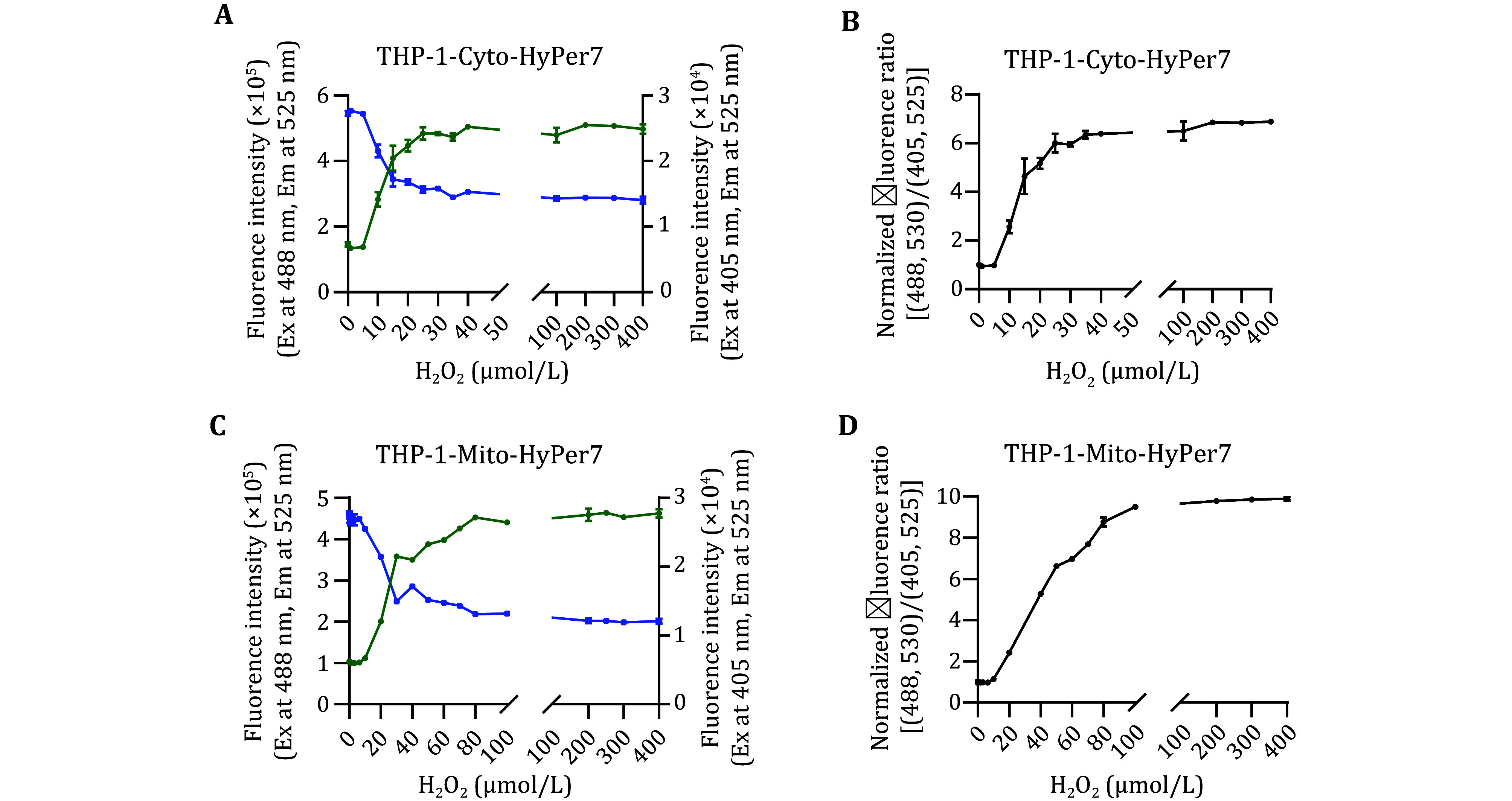
Dosage effects of the biosensors in response to exogenously adding H_2_O_2_. **A**, **B** Fluorescence intensity of THP-1-CytoHyPper7 cells (**A**) and THP-1-MitoHyPper7 cells (**B**) after being treated with H_2_O_2_ of gradient concentration upon 405 nm (purple) excitation and 488 nm (green). **C**,**D** Normalized fluorescence Ratio of THP-1-CytoHyPper7 cells (**C**) and THP-1-MitoHyPper7 cells (**D**) after being treated with H_2_O_2_ of gradient concentration

Having confirmed that HyPer7 is expressed and responsive, we performed a titration experiment to determine the minimal amount of exogenous H_2_O_2_ that is required to elicit a detectable probe response and the maximal detectable H_2_O_2_ concentration. We used flow cytometry to evaluate the variation in fluorescence intensity after co-incubation with H_2_O_2_. For THP-1-CytoHyPer7, the minimal detectable amount of exogenous H_2_O_2_ was 10 μmol/L, and F488 increased along with the climb of the H_2_O_2_ concentration when the concentration of exogenous H_2_O_2_ ranged from 10 to 40 μmol/L, and when the exogenous H_2_O_2_ concentration exceeds 40 μmol/L, the fluorescence intensity reached a plateau without further enhancement (Fig. 3A). For THP-1-MitoHyPer7, the minimal detectable amount of exogenous H_2_O_2_ was 10 μmol/L, and the detectable range was 10 to 100 μmol/L (Fig. 3B). To make it simpler, we used the normalized fluorescence ratio of F488 to F405 (F488/F405) to characterize the relative H_2_O_2_ compared to the untreated cells (Figs. 3C and 3D), the maximal F488/F405 of THP-1-CytoHyPer7 and THP-1-MitoHyPer7 reached 6.83 and 9.49, respectively.

### Monitor H_2_O_2_ level in response to chemotherapeutic agents

**Figure 4:**
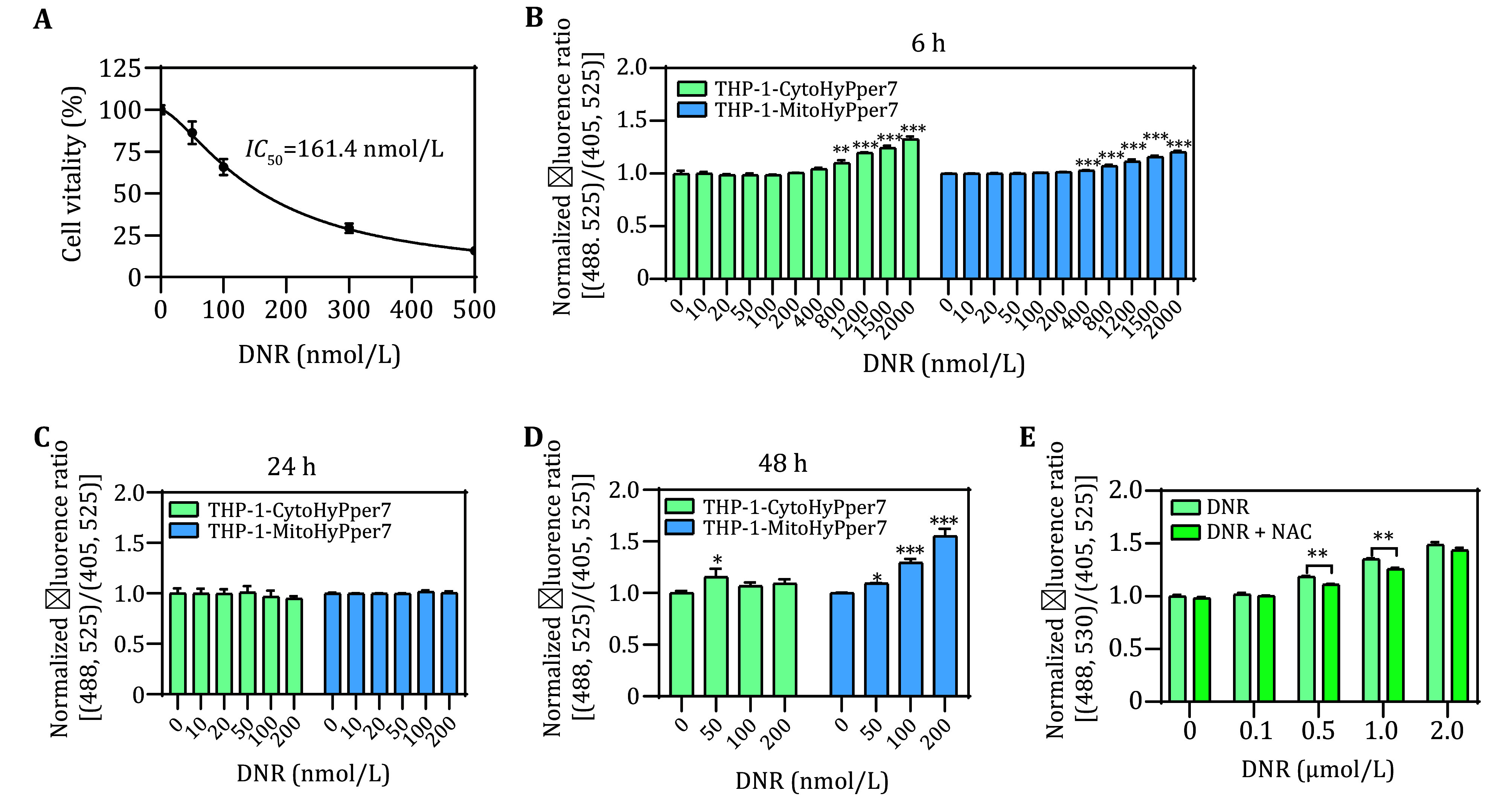
Detection of cytosolic and mitochondrial H_2_O_2_ perturbation after incubation with DNR. **A** Cell viability of THP-1 cells after incubation with DNR for 24 h. **B** Normalized fluorescence ratio of THP-1-CytoHyPper7 cells (green) and THP-1-MitoHyPper7 cells (blue) after being treated with DNR for 6 h. **C** Normalized fluorescence ratio of THP-1-CytoHyPper7 cells after being treated with DNR in the presence or absence of NAC for 6 h. **D**,**E** Normalized fluorescence ratio of THP-1-CytoHyPper7 cells (green) and THP-1-MitoHyPper7 cells (blue) after being treated with DNR for 24 h (**D**), and 48 h (**E**)

Most chemotherapeutic agents were reported to induce intracellular ROS accumulation through several mechanisms. Herein, we employed Daunorubicin (DNR) to monitor the dynamics of cytosolic and mitochondrial H_2_O_2_. It is well known that DNR plays its cytotoxicity by increasing intracellular ROS (Burt *et al.* 2019), however, the subcellular compartment of ROS generation was unclear. Our results from the CCK8 assay indicated the IC_50_ at 24 h was 161.4 nmol/L (Fig. 4A). By using the biosensors established in this study, we found out that in the dose range of 20 nmol/L to 200 nmol/L, the cytosolic and mitochondrial H_2_O_2_ remained unchanged after treated for 6 h and 24 h (Figs. 4B and 4C). Only a slight increase of F488/F405 was observed in the cytosolic H_2_O_2_ after treated for 48 h while a dramatic increase of 1.55 folds was observed in the mitochondrial H_2_O_2_ (Fig. 4D). It should be noted that in the 6-h experiment, dosages were increased up to 1.5 mmol/L. Results showed that the high doses of DNR increased cytosolic and mitochondrial H_2_O_2_ at the same time, and 1.5 mmol/L of DNR resulted in 1.33 folds and 1.21 folds of cytosolic and mitochondrial H_2_O_2_ compared with the untreated cells, respectively. These results indicated the long-term effects on H_2_O_2_ occurred primarily in mitochondria while the short-term and high-dose effects occurred in both mitochondria and cytosol. Moreover, the increase of cytosolic H_2_O_2_ induced by DNR could be partly reversed by N-acetylcysteine (NAC), indicating that the sensor cells were capable of sensing and detecting the attenuation of H_2_O_2_ induced by antioxidant (Fig. 4E).

Since ROS has been identified as one of the common mediators for chemo-resistance in leukemia (Trombetti *et al.* 2021), the constructed biosensors offer a powerful platform to monitor the dynamic of cytosolic and mitochondrial H_2_O_2_ in resistant or sensitive cells and to continuously monitor the adjustment of H_2_O_2_, which will be helpful to reveal the mechanism of chemotherapy resistance in leukemia.

### Monitor H_2_O_2_ level in response to nanozymes

**Figure 5:**
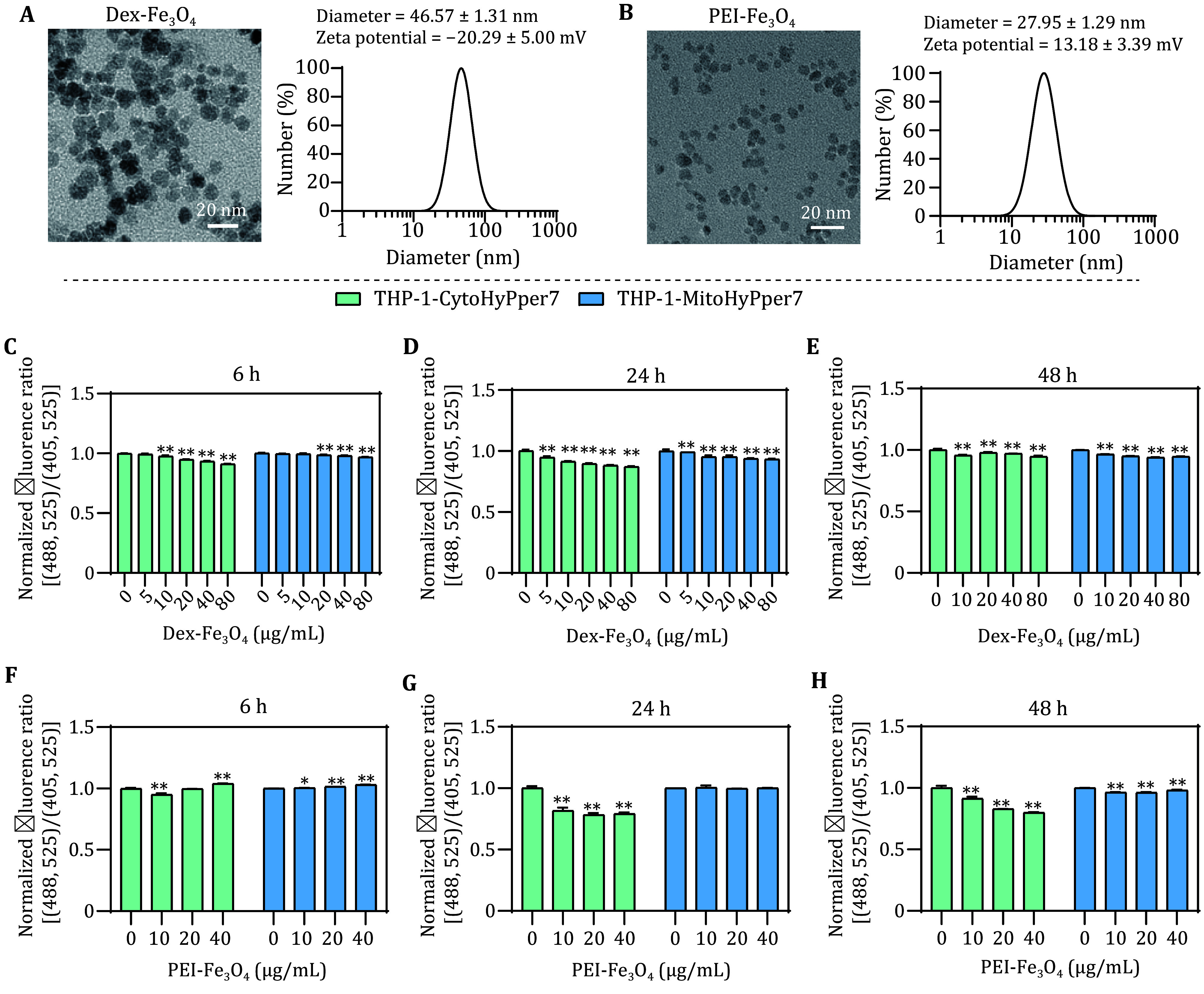
Detection of cytosolic and mitochondrial H_2_O_2_ perturbation after incubation with Dex-Fe_3_O_4_ and PEI-Fe_3_O_4_. **A**,**B** Size of Dex-Fe_3_O_4_ (**A**) and PEI-Fe_3_O_4_ (**B**) under TEM and the hydrodynamic diameter distributions in ddH_2_O measured by DLS. **C**−**E** Normalized fluorescence ratio of THP-1-CytoHyPper7 cells (green) and THP-1-MitoHyPper7 cells (blue) after being treated with Dex-Fe_3_O_4_ for 6 h (**C**), 24 h (**D**), and 48 h (**E**). **F**−**H** Normalized fluorescence ratio of THP-1-CytoHyPper7 cells (green) and THP-1-MitoHyPper7 cells (blue) after being treated with PEI-Fe_3_O_4_ for 6 h (**F**), 24 h (**G**), and 48 h (**H**)

Next, we applied the biosensors to monitor H_2_O_2_ dynamics after co-incubation with several nanozymes that have multi-enzyme properties to affect intracellular H_2_O_2_ concentrations. Fe_3_O_4_ nanoparticle was the first nanozyme reported with POD-like activity (Gao *et al.* 2007), and further investigations revealed its POD-like activity under the acidic environment (pH = 4.8) and CAT-like activity in neutral conditions (pH = 7.4) (Chen *et al.* 2012). Herein, by using the biosensors, we detected the dynamics of cytosolic and mitochondrial H_2_O_2_ after incubation with Fe_3_O_4_ nanoparticles coated with PEI (referred to as PEI-Fe_3_O_4_) and Dextran (referred to as Dex-Fe_3_O_4_). The diameters of both Fe_3_O_4_ nanoparticles were less than 20 nm under TEM (Figs. 5A and 5B). Their hydrodynamic diameters were 46.57 ± 1.31 nm and 27.95 ± 1.29 nm determined by DLS. Dex-Fe_3_O_4_ was negatively charged with zeta potentials of −20.29 ± 5.00 mV, while PEI-Fe_3_O_4_ was positively charged with zeta potentials of 13.18 ± 3.39 mV. After co-incubation with Dex-Fe_3_O_4_ for 6 h, cytosolic and mitochondrial H_2_O_2_ were scavenged at the same time and decreased further after 24 h (Figs. 5C−5E). PEI-Fe_3_O_4_ acted differently from Dex-Fe_3_O_4_. The variation in mitochondrial H_2_O_2_ was less than 1% after incubation with PEI-Fe_3_O_4_ for 6 h, which can be considered unchanged (Fig. 5F). At the same time, the variation of cytosolic H_2_O_2_ was uncommon, that was, it decreased by 5% upon being treated with 10 mg/L PEI-Fe_3_O_4_, while as the concentration climbed to 20 mg/L and 40 mg/L, H_2_O_2_ gradually rose and became comparable to the control group in the 40 mg/L PEI-Fe_3_O_4_ group (Fig. 5F). After co-incubation for 24 h, the cytosolic H_2_O_2_ was diminished compared to the untreated cells (Fig. 5G). As the incubation time was extended to 48 h, the concentration of mitochondrial H_2_O_2_ decreased too (Fig. 5H). Therefore, it is plausible to consider that the overall H_2_O_2_ was attributed to both the nanozyme’s properties and the surface modification of the nanoparticles.

Surface modification-dependent effects (Fig. 5) highlight the need for quick screening for the intracellular effects of nanozymes, especially those that could modulate ROS. The biosensors provide a standardized system to guide surface engineering to minimize unintended ROS modulation.

**Figure 6:**
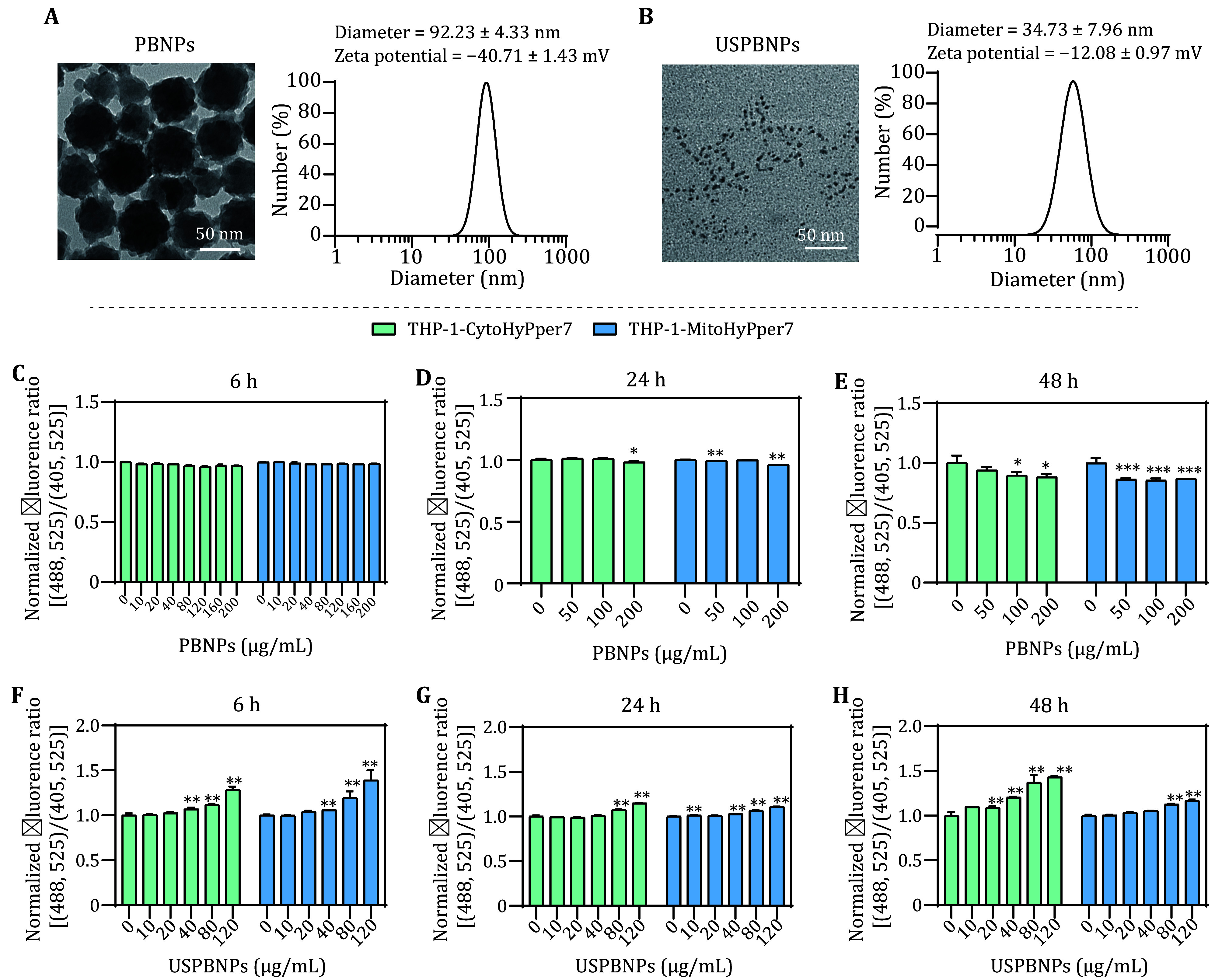
Detection of cytosolic and mitochondrial H_2_O_2_ perturbation after incubation with PBNPs and USPBNPs. **A**,**B** Size of PBNPs (**A**) and USPBNPs (**B**) under TEM and their hydrodynamic diameter distribution in ddH_2_O determined by DLS. **C**−**E** Normalized fluorescence ratio of THP-1-CytoHyPper7 cells (green) and THP-1-MitoHyPper7 cells (blue) after being treated with PBNPs for 6 h (**C**), 24 h (**D**), and 48 h (**E**). **F**−**H** Normalized fluorescence ratio of THP-1-CytoHyPper7 cells (green) and THP-1-MitoHyPper7 cells (blue) after being treated with USPBNPs for 6 h (**F**), 24 h (**G**), and 48 h (**H**)

PBNPs were reported for their ability of scavenging ROS both in tubes as well as in cells (Zhang *et al.* 2016). In this study, we detect the intracellular effects of two PBNPs with different diameters by using the established biosensors. The Prussian Blue nanoparticles exhibit a sub-spherical shape under TEM with a diameter of about 60 nm**,** and the hydrodynamic diameter was determined to be 92.23 ± 4.33 nm (Fig. 6A). Ultrasmall Prussian Blue nanoparticles exhibit a cluster-like shape under TEM with a diameter of about 5 nm**,** and the hydrodynamic diameter was determined to be 34.73 ± 7.96 nm (Fig. 6B). PBNPs and USPBNPs were both negatively charged with zeta potentials of −12.08 ± 0.97 mV and −40.71 ± 1.43 mV, respectively. We found out that the PBNPs didn’t change cytosolic or mitochondrial H_2_O_2_ after 6 h incubation and eventually scavenged H_2_O_2_ by 13% after being treated for 48 h (Figs. 6C−6E). However, it is interesting to see that USPBNPs exhibited different overall effects on the intracellular H_2_O_2_. The cytosolic and mitochondrial H_2_O_2_ was elevated upon co-incubation with USPBNPs (Figs. 6F−6H), which suggested that the particle size of nanozyme also played a role in regulating intracellular H_2_O_2_, though the high POD-like and CAT-like activities were demonstrated in USPBNPs over PBNPs.

**Figure 7:**
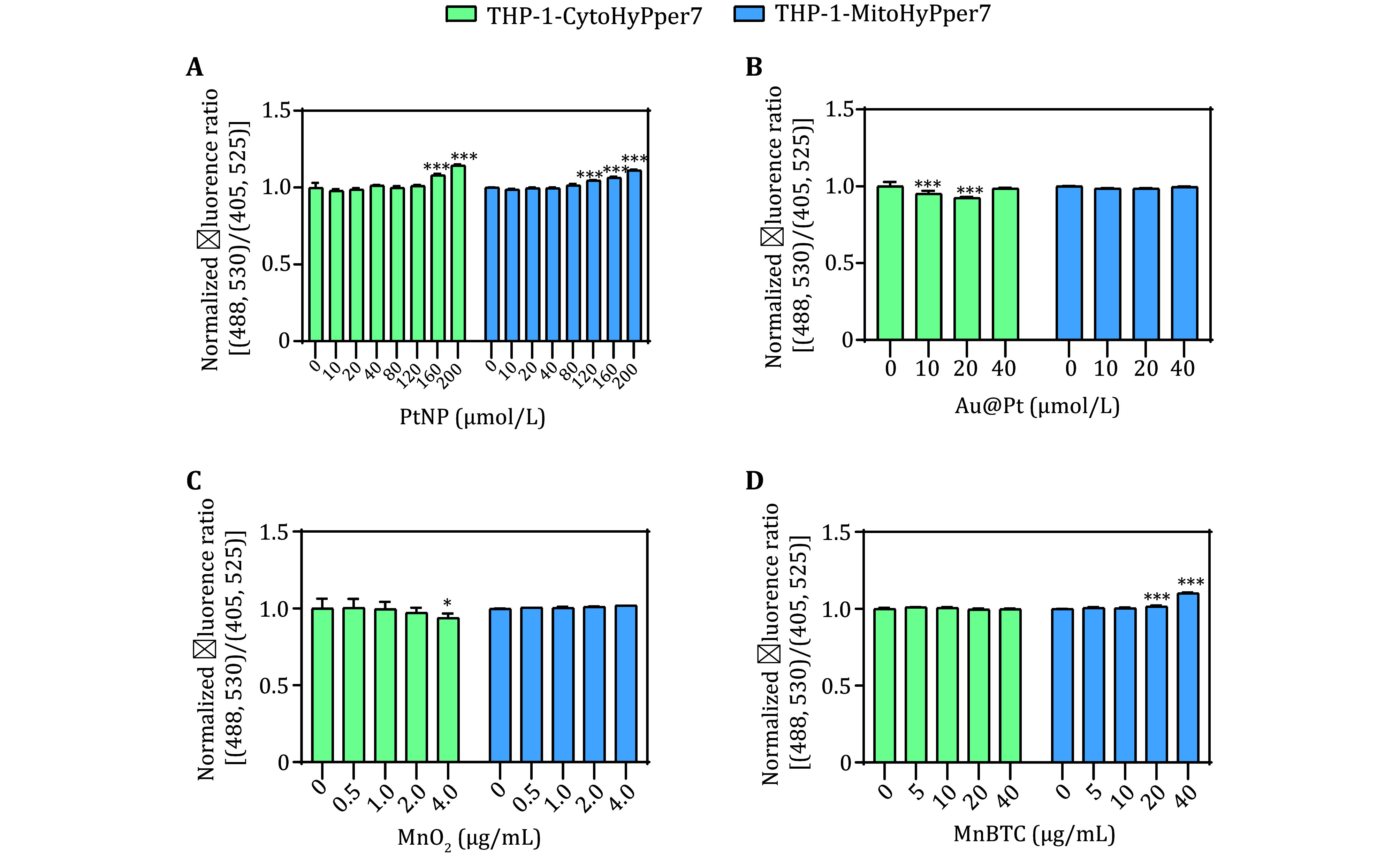
Normalized fluorescence ratio of THP-1-CytoHyPper7 cells and THP-1-MitoHyPper7 cells after incubation with PtNPs (**A**), Au@Pt (**B**), MnO_2_ (**C**), MnBTC (**D**) nanozymes for 24 h

Mn-based and Pt-based nanozymes were also detected with the biosensors. After co-incubation for 24 h, the PtNPs caused an increase in both cytosolic and mitochondrial H_2_O_2_. MnO_2_ and Au@Pt decreased cytosolic H_2_O_2_ without changing mitochondrial H_2_O_2_, while MnBTC increased mitochondrial H_2_O_2_ without changing cytosolic H_2_O_2_ (Fig. 7).

## CONCLUSION

In summary, this work constructed the genetically encoded fluorescent sensors CytoHyper7 and MitoHyper7 to detect cytosolic and mitochondrial H_2_O_2_ levels. The performance of the sensors was characterized by fluorescent spectroscopy, and the responses to chemotherapeutics DNR and nanozymes of PBNPs with different diameters and iron oxide nanoparticles with different surface modifications were revealed. Results obtained by using the biosensors indicated that the particle size of PBNPs and surface modification of Fe_3_O_4_ play critical roles in their intracellular effects on the aspect of H_2_O_2_ modulation.

## Conflict of interest

Tao Wang, Mengfan Yu, Chenshuo Ren, Fan Yang, Tao Wen, Xian-En Zhang, Haoan Wu, Yu Zhang, Dianbing Wang and Haiyan Xu declare that they have no conflict of interest.
